# Screen Time and Behavioral Correlates Among Children in the United Arab Emirates: A Cross-Sectional Study

**DOI:** 10.7759/cureus.106417

**Published:** 2026-04-04

**Authors:** Ola M Tarkhoun, Maryam O Al-Tamo, Tasnime M Brinsi, Rooa F Bin Kuwaiyer, Aya B Kanani, Salsabila M Brinsi, Yusuf Parvez

**Affiliations:** 1 College of Medicine, Dubai Medical University, Dubai, ARE; 2 College of Medicine, University of Sharjah, Sharjah, ARE; 3 General Practice, Thumbay Hospital Fujairah, Fujairah, ARE

**Keywords:** bedroom screen use, behavioural problems, child's screen time, parental mediation, united arab emirates (uae)

## Abstract

Background: The rapid digitization of childhood has intensified concerns over excessive screen exposure and its behavioral consequences. In the United Arab Emirates (UAE), where digital access begins early and is near-universal, evidence on how screen use relates to behavioral symptoms remains limited. This study explored associations between daily screen time, behavioral concerns, and parental mediation among children aged 1-15 years in the UAE.

Methods: A cross-sectional survey was conducted between March and August 2025 across two healthcare centers and through community referral networks. A total of 405 parents of children aged 1-15 years completed a culturally adapted bilingual online questionnaire. Variables included average screen hours, device use locations, behavioral symptoms, and parental regulation strategies. Behavioral symptoms were measured using an eight-item checklist. Negative binomial regression was used to assess predictors of symptom burden.

Results: Children averaged 4.05 screen hours/day (SD = 2.34), with usage increasing significantly with age. Adolescents aged 11-15 years averaged 5.05 hours/day. Frequently reported behavioral symptoms were reduced attention (197, 48.6%), decreased physical activity (169, 41.7%), and irritability/tantrums (166, 41.0%). Each additional hour of screen time was associated with a 3% increase in behavioral symptom rate (IRR = 1.031, 95% CI: 1.001-1.062, p = 0.041). Bedroom screen use independently predicted a 30% higher symptom rate (IRR = 1.300, 95% CI: 1.130-1.495, p < 0.001). Although parents employed multiple mediation strategies, the number of strategies used did not independently reduce screen time or behavioral burden after adjustment.

Conclusions: Screen time duration and context, particularly bedroom access, were significant independent predictors of behavioral symptoms among children in the UAE. Despite high parental awareness, mediation strategies showed limited independent efficacy. Public health guidance should move beyond time-based recommendations and emphasize screen-free environments and consistent digital parenting.

## Introduction

Children’s daily routines increasingly revolve around televisions, tablets, smartphones, and other connected devices that shape communication, learning, and leisure. Global surveillance indicates that only 24.7% of children younger than two years meet the recommendation of avoiding screen exposure entirely, and only 35.6% of children aged 2-5 years adhere to a limit of ≤1 hour/day [1]. A growing body of evidence links excessive or unstructured screen use with specific behavioral concerns, including attentional problems, irritability, sleep disturbance, and reduced physical activity [2,3].

Despite this expanding international literature, empirical evidence from rapidly modernizing Middle Eastern settings remains limited. The UAE is characterized by near-universal digital connectivity, early device access among children, and strong family and communal structures that may influence both exposure patterns and parental mediation. National data indicate that 86% of children in the UAE regularly engage in digital activities [4]. In addition, only 26% of secondary-school students meet the recommended recreational screen-time threshold of ≤2 hours/day, suggesting a widespread sedentary digital lifestyle [5,6]. Prolonged screen exposure has also been associated with speech and language delay among Emirati toddlers, highlighting the developmental salience of early device use [7].

Important knowledge gaps remain. Existing regional studies often focus on broad categories of use or physical outcomes, leaving a need to quantify the relationship between screen time and a wider range of specific behavioral symptoms in the UAE context. The role of screen-use context, such as bedroom access and patterns of parental supervision, is also underexplored quantitatively. Qualitative work among Emirati mothers suggests reliance on restrictive and co-viewing strategies when regulating children’s YouTube use, but also difficulty maintaining consistent supervision [8]. Similar evidence from Bahrain suggests that screen use may interact with family routines and emotional responses when devices are removed [9].

Two conceptual frameworks informed the interpretation of this study. The displacement hypothesis proposes that screen time can crowd out developmentally enriching activities such as play, sleep, and physical interaction [10]. Social learning theory further suggests that children learn patterns of behavior through repeated interactions within their environment, including parental responses to digital media use [11,12]. In the UAE’s distinctive family setting, these mechanisms may interact in ways that shape both exposure and behavioral outcomes.

This study aimed to assess the relationship between daily screen time and behavioral changes among children aged 1-15 years in the UAE. Specifically, it sought to (1) determine average daily screen exposure across key sociodemographic groups, (2) identify specific behavioral symptoms associated with increased daily screen exposure, (3) assess parental perceptions of screen time and the relationship between mediation strategies and children’s screen habits, and (4) identify the independent socio-behavioral and contextual predictors of behavioral symptom burden.

## Materials and methods

Study design and setting

This cross-sectional study was conducted in the UAE between March and August 2025. Data were collected using an anonymous, self-administered online questionnaire. Recruitment occurred in selected primary healthcare centers, Rashid Hospital (Dubai) and Thumbay Hospital (Fujairah), where trained staff displayed a study QR code in waiting areas. Additional participants were reached through direct sharing of the survey link within relatives’ and caregivers’ personal networks, resulting in a convenience sample supplemented by snowball referral. The survey was available in both Arabic and English. No advertisements were used, and no counseling or intervention was provided at any stage.

Participants and eligibility criteria

Eligible participants were parents or legal guardians of children aged 1-15 years residing in the UAE at the time of the survey. Respondents had to be 18 years or older. Parents of children with major neurodevelopmental disorders, including autism spectrum disorder and diagnosed developmental delays, were excluded because screen-behavior relationships may differ substantially in these populations. Recruitment occurred through two routes: clinic-based recruitment through QR-code access in participating facilities and community referral through direct participant-to-participant sharing of the survey link within personal networks. Invitation counts, QR-scan rates, and the reach of community sharing were not tracked prospectively; therefore, a response rate could not be calculated, and platform-specific dissemination details could not be reconstructed retrospectively.

Sampling approach and sample size

A non-probability sampling strategy combining convenience sampling and referral-based recruitment was used because a comprehensive sampling frame for UAE parents was not available. Although this approach limits generalizability, it is frequently used in behavioral research conducted in healthcare settings and community networks. The minimum required sample size was 384 participants, calculated using Cochran’s formula for cross-sectional surveys assuming a 95% confidence level, 5% margin of error, and maximum variability (p = 0.50). The achieved sample of 405 exceeded this threshold.

Survey development and measures

The questionnaire assessed screen time, screen-use context, behavioral symptoms, and parental mediation strategies. Items were developed from the screen-time literature and adapted for cultural relevance in the UAE. Behavioral symptoms were captured using a study-developed survey item asking, “Have you noticed any behavioral changes in your child related to screen time? Please specify (select all that apply).” The eight analyzed response options were increased irritability or tantrums, decreased attention span, increased hyperactivity, decreased physical activity, difficulty sleeping or nightmares, social withdrawal or reduced interaction with family, increased aggression, and anxiety or mood swings. For analysis, each symptom option was coded dichotomously (selected = yes; not selected = no), yielding an eight-item behavioral checklist. The checklist was piloted with 15 caregivers to assess clarity and face validity, and minor wording adjustments were made.

Translation and reliability

The English draft was forward-translated into Arabic by a bilingual researcher and independently back-translated into English. Discrepancies were resolved by consensus. The Arabic version then underwent cognitive testing with a separate group of 15 caregivers to confirm comprehension. Internal consistency analysis of the eight dichotomous behavioral symptom items demonstrated acceptable reliability (Cronbach’s α = 0.694, 95% CI: 0.647-0.741). Item-rest correlations ranged from 0.238 to 0.497, and alpha-if-item-deleted values (0.639-0.696) did not support removing any item.

A tabular summary of the questionnaire structure is provided in the Appendices.

Variables and operational definitions

Daily screen time was the primary exposure and was treated as a continuous parent-reported measure of average hours/day. For descriptive contrasts, prolonged exposure was defined as >4 hours/day. Screen activities were recorded as binary indicators for videos, gaming, social media, and educational apps. Screen locations were recorded as binary indicators for the living room, bedroom, during meals, at school, or in the car/travel. Bedroom screen use was coded as yes/no. A multi-location score captured the number of distinct screen-use locations (range 0-5). The behavioral symptom composite was defined as the sum of the eight symptom items (range 0-8). Parental mediation was operationalized as the number of strategies used, including setting time limits, encouraging alternative activities, co-viewing, and parental control apps.

Data collection and quality control

Data were collected electronically through an online survey platform. Mandatory fields prevented progression with missing core responses. No personally identifying information was collected. Because IP- or device-based duplicate detection was not activated, multiple entries from the same household cannot be ruled out; however, no incentives were offered. Responses were exported to Excel (Microsoft Corp., Redmond, WA, USA) and then IBM SPSS Statistics for Windows (IBM Corp., Armonk, NY, USA)/JASP (University of Amsterdam, Amsterdam, Netherlands) for cleaning and analysis. Data checks included screening for out-of-range values, verifying logical consistency across items, and removing incomplete records for which the behavioral composite could not be calculated. Missing data were minimal (<1%), and complete-case analysis was used. No imputation was performed.

Statistical analysis

Analysis was performed using JASP version 0.95.4 [13,14]. Descriptive statistics summarized demographic variables, daily screen time, activities, and behavioral symptoms. Group comparisons used independent t-tests and one-way ANOVA with Levene’s test for homogeneity of variance; Welch’s correction was applied when needed. Associations between screen time and behavioral symptoms were examined using Pearson correlation. Because the behavioral symptom composite was a count outcome with overdispersion, multivariable modeling used negative binomial regression via the glm.nb() function from the MASS package in R (R Foundation for Statistical Computing, Vienna, Austria, https://www.R-project.org/) to estimate incidence rate ratios (IRRs) and 95% confidence intervals (CIs). Final models adjusted for child age group, gender, parental nationality, household income, and bedroom screen exposure. Statistical significance was defined as p < 0.05 (two-tailed).

Bias and ethical considerations

Potential sources of bias included selection bias due to non-probability sampling, information bias from parent-reported screen use and behavior, the possibility of duplicate responses because de-duplication was not implemented, single-informant bias, and measurement bias because the behavioral checklist was not a validated psychometric scale. The study received ethical approval from the Dubai Medical University Research Ethics Committee (REC/DMCG/AY24-25/S-34). Participation was voluntary, and electronic informed consent was obtained before the survey began. All procedures were conducted in accordance with the Declaration of Helsinki.

## Results

A total of 405 parents completed the survey. Results are presented according to the four study objectives.

Objective 1: daily screen exposure across sociodemographic groups

Across the full sample, mean daily screen exposure was 4.05 hours (SD = 2.34; 95% CI: 3.82-4.28). As shown in Table 1, screen time increased steadily with age: children aged 1-3 years averaged 2.57 hours/day, those aged 4-6 years averaged 3.62 hours/day, those aged 7-10 years averaged 3.87 hours/day, and adolescents aged 11-15 years averaged 5.05 hours/day. Differences by gender were small, and expatriate and UAE-national families reported similar exposure levels. Household income showed a slight but non-significant gradient, with higher-income families reporting marginally more screen use.

**Table 1:** Sample characteristics and mean daily screen time (N = 405) Note: Group comparisons for mean screen time were calculated using one-way ANOVA for child age group and household income and independent-samples t-tests for child gender and parental nationality.

Variable	n (%)	Mean screen time (hours/day)	SD	Test statistic	p-value
Child age group				F = 16.14	<0.001
1-3 years	41 (10.1%)	2.57	2		
4-6 years	100 (24.7%)	3.62	2.14		
7-10 years	137 (33.8%)	3.87	2.16		
11-15 years	127 (31.4%)	5.05	2.42		
Child gender				t = 0.58	0.562
Male	227 (56.0%)	4.11	2.4		
Female	178 (44.0%)	3.97	2.28		
Parental nationality				t = -0.21	0.833
Expatriate	303 (74.8%)	4.03	2.41		
UAE national	102 (25.2%)	4.09	2.16		
Household income				F = 0.70	0.55
<5,000 AED	37 (9.1%)	3.69	2.43		
5,000-10,000 AED	95 (23.5%)	3.98	2.11		
10,000-20,000 AED	115 (28.4%)	3.95	2.51		
>20,000 AED	158 (39.0%)	4.23	2.35		
Overall sample	405 (100%)	4.05	2.34		

**Figure 1:**
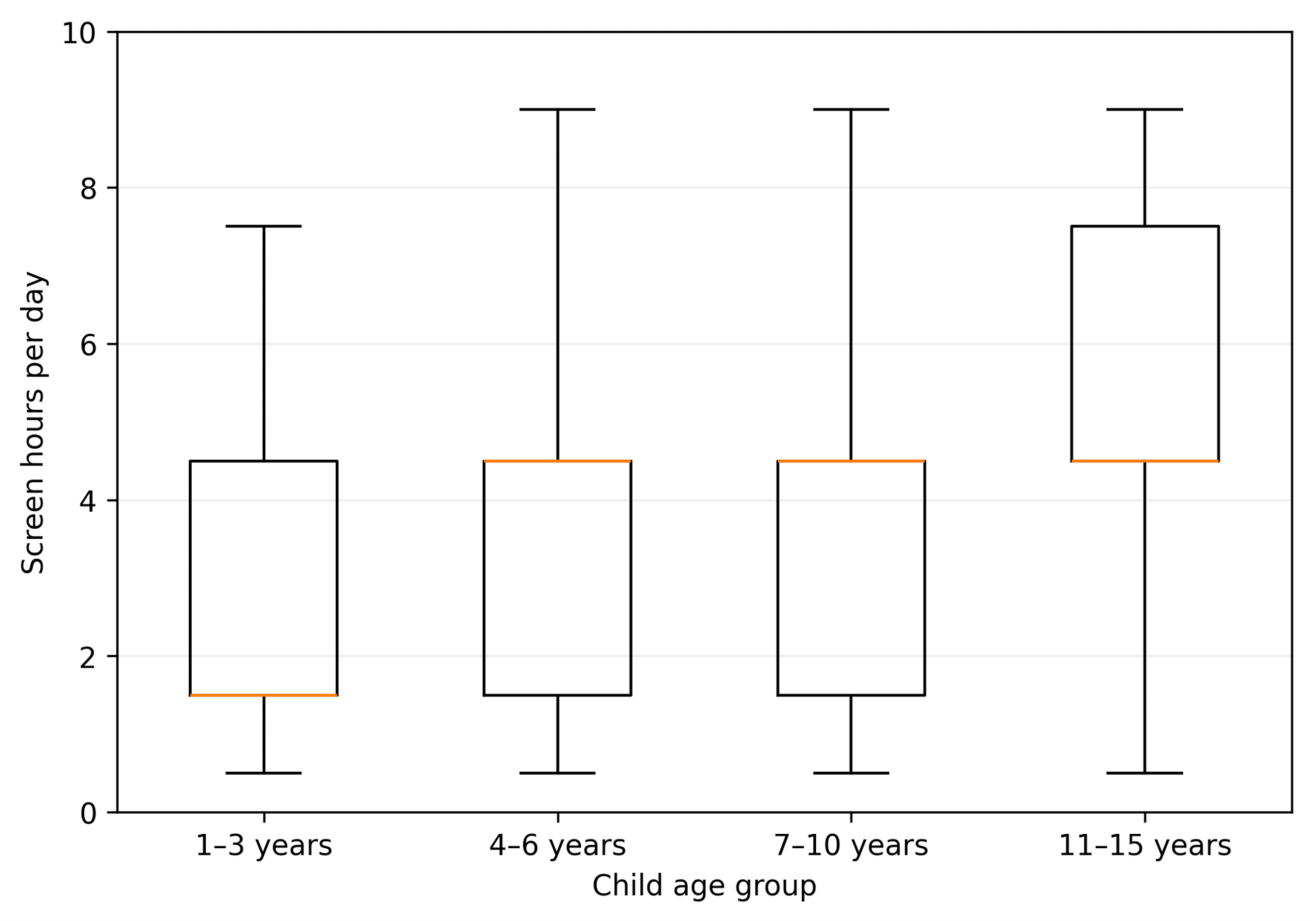
Distribution of daily screen time (hours) stratified by child age group

Figure 1 shows the distribution of daily screen hours by child age group. The distribution was right-skewed across groups, with a clear shift toward higher exposure among adolescents. Secondary subgroup analyses by gender, nationality, and household income did not reveal statistically meaningful differences in daily screen exposure (all p > 0.05).

Objective 2: behavioral correlates of daily screen time

**Figure 2:**
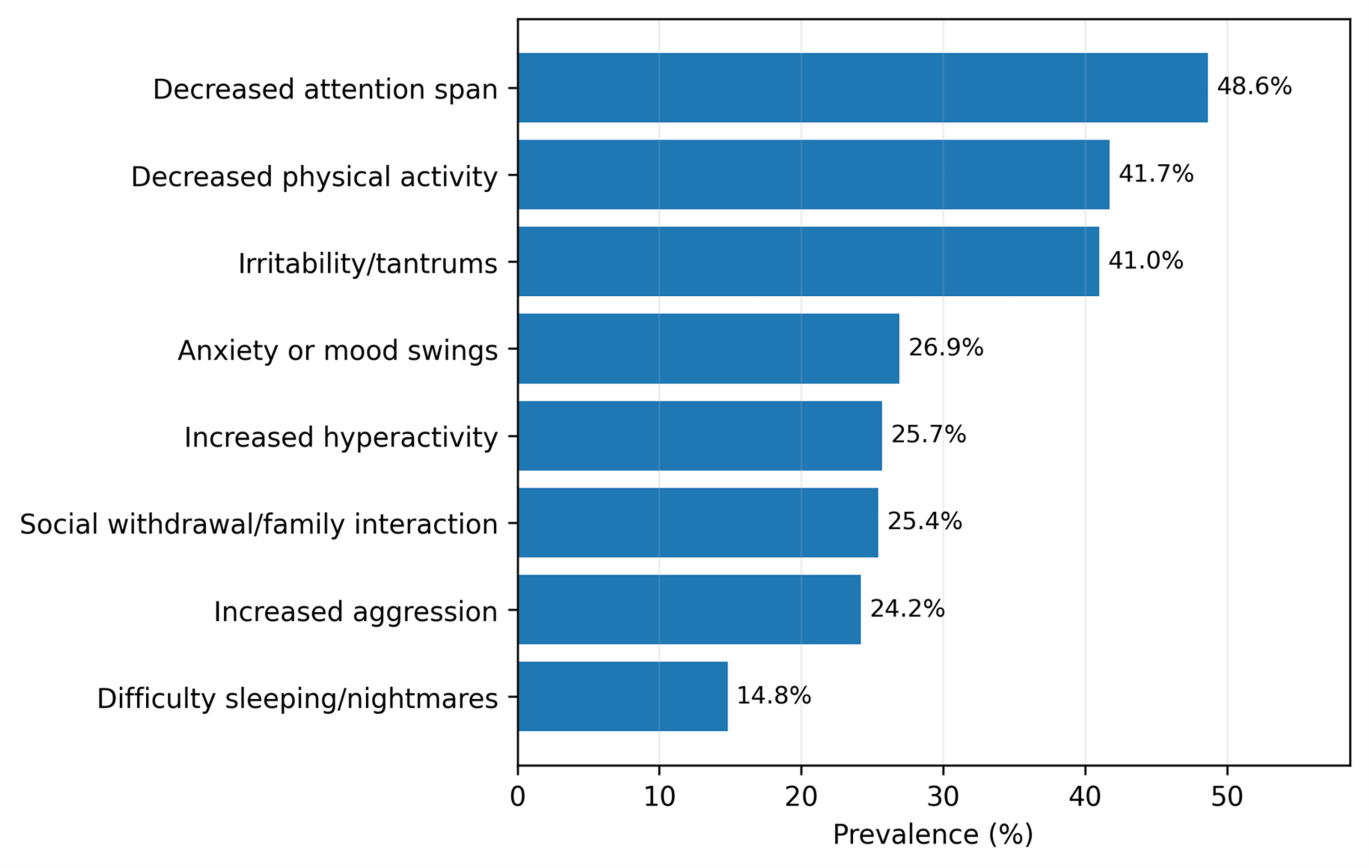
Prevalence of parent-reported behavioral symptoms in the study sample (N = 405)

Behavioral symptom prevalence is summarized in Figure 2. The most frequently reported concerns were decreased attention span (197, 48.6%), decreased physical activity (169, 41.7%), and irritability/tantrums (166, 41.0%). Sleep-related problems were the least common (60, 14.8%).

**Figure 3:**
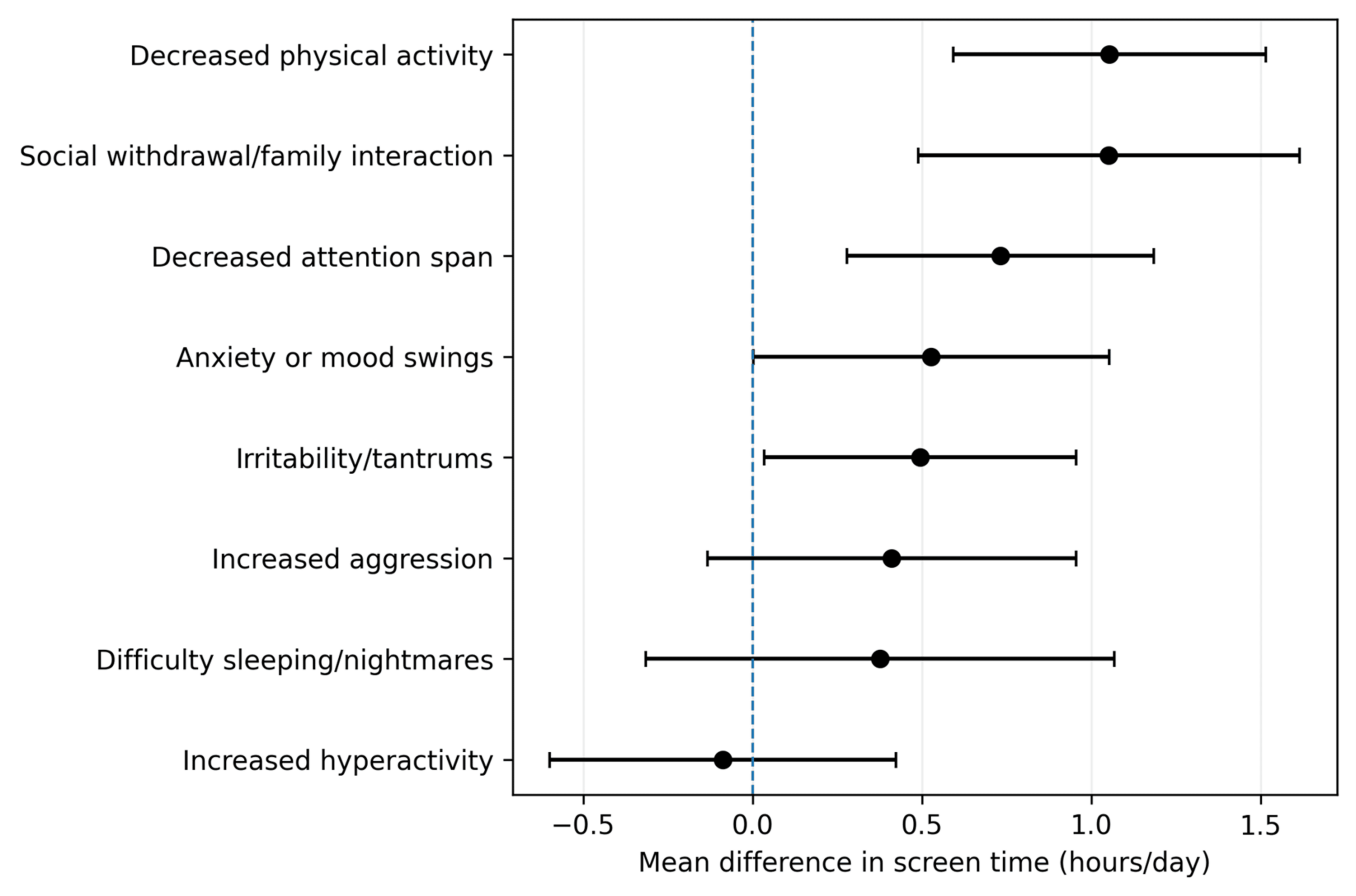
Mean differences in daily screen time between children with and without specific behavioral symptoms. Error bars represent 95% confidence intervals

Higher screen exposure was associated with decreased physical activity (+1.05 h/day; 95% CI: 0.61-1.49), social withdrawal/family interaction (+1.05 h/day; 95% CI: 0.55-1.54), decreased attention span (+0.73 h/day; 95% CI: 0.27-1.19), anxiety or mood swings (+0.53 h/day; 95% CI: 0.01-1.05), and irritability/tantrums (+0.50 h/day; 95% CI: 0.03-0.97). Differences for aggression, sleep difficulties, and hyperactivity were not statistically significant. Overall, children with emotional-behavioral symptoms tended to have higher daily screen exposure than children without such symptoms (Figure 3).

Objective 3: parental perceptions and mediation strategies

**Figure 4:**
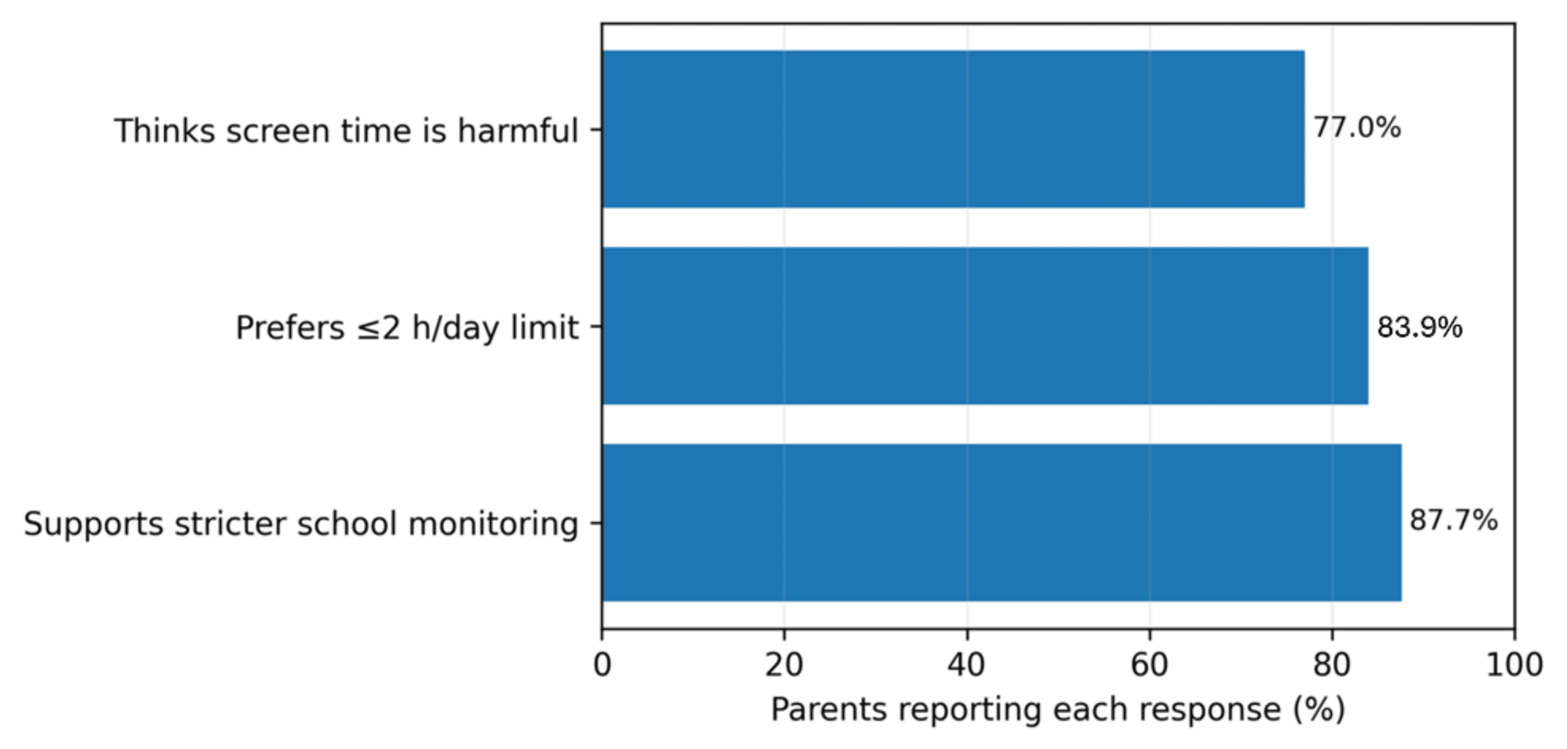
Parental perceptions of screen time among children

Parental attitudes toward screen use are summarized in Figure 4. Most parents (312, 77.0%) believed that screen time negatively affects their child; 340 (83.9%) preferred a daily limit of two hours or less; and 355 (87.7%) supported stricter monitoring of screen use in schools.

Parents who believed that screen time negatively affects their child reported higher behavioral symptom scores than parents who did not hold that belief. Because the behavioral composite deviated significantly from normality (Shapiro-Wilk W = 0.865, p < 0.001), a Mann-Whitney U test was used; it showed significantly higher ranks in the “Yes” group than in the “No” group (U = 7,771, p < 0.001; rank-biserial r = -0.384).

Children whose parents believed that screen time negatively affects their child had slightly higher mean daily screen exposure (4.04 hours/day, SD = 2.35, n = 312) than children whose parents did not hold this belief (3.43 hours/day, SD = 2.02, n = 36), but this difference was not statistically significant (Mann-Whitney U = 6,360.5, p = 0.157; rank-biserial r = −0.13).

Parents used multiple strategies to manage their children’s screen time (mean = 2.51 strategies, range = 1-4). As shown in Table 2, the most common strategy was setting time limits (357, 88.1%), followed by encouraging alternative activities (303, 74.8%).

**Table 2:** Frequency of parental mediation strategies (N = 405) Note: Descriptive frequencies only; no hypothesis test was applied to this table. Accordingly, the test statistic and p-value columns are listed as not applicable (NA).

Strategy	n	%	Test statistic	p-value
Setting time limits	357	88.10%	NA	NA
Encouraging alternative activities	303	74.80%	NA	NA
Using parental control apps	179	44.20%	NA	NA
Co-viewing content with the child	179	44.20%	NA	NA

Spearman’s rank-order correlation showed a weak but statistically significant negative association between the number of parental mediation strategies and children’s daily screen time (ρ = -0.108, p = 0.030, 95% CI: -0.206 to -0.009). In a multivariable linear regression model adjusting for age group, parental nationality, household income, and parental perceptions, the number of strategies was no longer a significant predictor of daily screen time (β = -0.185, p = 0.09). Child age remained the strongest predictor, with younger children reporting substantially lower exposure than adolescents.

**Table 3:** Statistical associations for parental perceptions and mediation strategies (N = 405) Note: Mann-Whitney U tests were used for non-parametric yes/no group comparisons; Spearman’s rank-order correlation was used for the unadjusted association between strategy count and daily screen exposure; and the adjusted coefficient for strategy count is reported from the multivariable linear regression model using the t statistic. “Not sure” responses were excluded from the yes/no parental perception comparisons.

Analysis	Groups/variables	n/model	Effect estimate	Test statistic	p-value
Behavioral symptom score by parental perceived harm	Yes vs no	Yes n = 312; no n = 36	Rank-biserial r = -0.384	Mann-Whitney U = 7,771	<0.001
Daily screen exposure by parental perceived harm	Yes vs no	Yes n = 312; no n = 36	Rank-biserial r = -0.13	Mann-Whitney U = 6,360.5	0.157
Strategy count vs daily screen exposure	Continuous association	N = 405	Spearman ρ = -0.108 (95% CI: -0.206 to -0.009)	Spearman ρ = -0.108	0.030
Adjusted association of strategy count with daily screen exposure	Multivariable linear regression	N = 405	β = -0.185	t = -1.70	0.090

Objective 4: multivariable predictors of behavioral symptoms

A fully adjusted negative binomial regression model examined independent predictors of the behavioral symptom composite. Screen time and bedroom screen use remained significant after adjustment for age group, gender, parental nationality, and household income (Table 4).

**Table 4:** Predictors of behavioral symptom counts: adjusted IRRs from negative binomial regression Reference categories are shown in parentheses. Wald z statistics and p-values were obtained from the adjusted negative binomial regression model. Model adjusted for screen time, bedroom screen use, age group, gender, parent nationality, and household income. p < 0.05 was considered statistically significant. IRR: incidence rate ratio.

Predictor	IRR	95% CI	Wald z	p-value
Screen time (hours/day)	1.031	1.001-1.062	2.04	0.041
Bedroom screen use (yes)	1.3	1.130-1.495	3.67	<0.001
Age group (ref = 11-15 years)				
4-6 years	1.077	0.901-1.286	0.82	0.414
7-10 years	1.029	0.875-1.210	0.34	0.731
1-3 years	0.725	0.544-0.955	-2.24	0.025
Gender (ref = male)				
Female	1.004	0.883-1.141	0.06	0.95
Parent nationality (ref = expatriate)				
UAE national	1.111	0.953-1.293	1.36	0.175
Household income (ref = >20,000 AED)				
<5,000 AED	0.9	0.698-1.149	-0.83	0.405
10,000-20,000 AED	0.914	0.779-1.071	-1.11	0.267
5,000-10,000 AED	0.941	0.789-1.119	-0.69	0.492

Each additional hour of daily screen exposure was associated with a 3% higher symptom rate (IRR = 1.031, 95% CI: 1.001-1.062, p = 0.041). Children with screen devices in their bedroom had 30% higher symptom levels than children without bedroom access (IRR = 1.300, 95% CI: 1.130-1.495, p < 0.001). Compared with adolescents, children aged 1-3 years had significantly lower symptom rates (IRR = 0.725, 95% CI: 0.544-0.955, p = 0.025). No significant associations were observed for gender, parental nationality, or household income.

## Discussion

Interpretation of key findings

This study provides one of the first quantitative assessments of screen time, its behavioral correlates, and parental mediation strategies among children aged 1-15 years in the UAE. Four key findings emerged: screen exposure was high and increased with age; both total screen time and bedroom access independently predicted greater behavioral symptom burden; parental concern was widespread, yet the number of mediation strategies had only a weak association with lower screen time; and sociodemographic characteristics were not significant predictors of behavioral symptoms after accounting for screen-related variables.

The finding that each additional hour of daily screen time was associated with a 3% increase in behavioral symptoms is consistent with prior meta-analytic evidence linking screen exposure to internalizing and externalizing problems in children [15]. Although a 3% increase per hour may appear modest, the difference between a child with two hours of exposure and a child with six hours of exposure corresponds to an estimated 12% higher symptom burden. The strongest predictor in the final model was bedroom screen access, which was associated with a 30% higher symptom rate. This suggests that the context of use may be as important as the duration of use itself. However, because the study was cross-sectional, this association should not be interpreted causally, and a bidirectional process cannot be excluded. One plausible explanation is that bedroom devices may displace sleep and other developmentally protective routines, thereby contributing to irritability, attention problems, and mood changes [10,16].

The relationship between parental mediation and outcomes appeared complex. The number of strategies used showed only a weak unadjusted association with lower screen exposure and did not remain significant after adjustment. One possible interpretation is that strategy count may reflect reactive mediation: parents facing more problematic screen use or more pronounced behavioral symptoms may deploy more tactics, but without necessarily improving outcomes. This interpretation is compatible with prior work suggesting that the quality and consistency of parental mediation, rather than its mere quantity, may be the more meaningful determinant of child digital behavior [17].

It is also notable that once screen exposure and bedroom access were taken into account, household income and parental nationality were not independently associated with behavioral symptoms. This suggests that screen-related behaviors may be more proximal determinants of symptom burden than broader demographic characteristics in this sample. The lower symptom burden observed among children aged 1-3 years likely reflects both lower independence in device use and age-related differences in content and routines.

Implications for practice and policy

These findings translate into several practical implications. First, counseling for families should move beyond time-based limits alone and routinely address context, particularly bedroom screen access and screen use during routine family activities. Second, parental guidance should emphasize proactive, consistent family media plans rather than ad hoc or purely reactive restrictions. Third, the strong parental support for stricter school monitoring (355, 87.7%) suggests that school-based public health messaging about screen-free bedrooms, balanced routines, and physical activity may be well received in the UAE setting.

Strengths and limitations

Strengths of this study include its focus on an under-researched regional context, a sample size that exceeded the minimum calculated requirement, a bilingual instrument adapted for local use, and regression modeling appropriate for an overdispersed count outcome. Several limitations should nevertheless be acknowledged. First, the cross-sectional design precludes causal inference and leaves open reverse-causation or bidirectional explanations, including the possibility that screens may sometimes be used reactively to manage pre-existing behavioral dysregulation. Second, the non-probability convenience and referral-based sampling strategy limits generalizability and may have overrepresented families who were easier to reach through clinic and personal-network recruitment. Third, all key measures relied on parent report, introducing single-informant and recall bias. Finally, the study-developed behavioral checklist showed acceptable but moderate internal consistency (Cronbach’s α = 0.694) and should not be interpreted as equivalent to a validated clinical instrument. Future research should use longitudinal designs, incorporate validated behavioral tools, prospectively document recruitment reach, and distinguish more clearly between proactive and reactive parental mediation styles.

## Conclusions

In this cross-sectional sample of UAE children, screen time and its context, particularly bedroom access, were associated with behavioral symptoms independent of sociodemographic factors. High levels of parental concern, combined with the limited independent effect of mediation strategy quantity, underscore the need for more nuanced public health guidance and clinical support. These findings support attention to context-based family rules, including consideration of screen-free bedrooms, while causal and interventional questions should be tested in longitudinal studies.

## Appendices

Questionnaire structure and source note

This appendix reformats the study questionnaire into tables with separate English/original and Arabic/translated columns, in line with the journal’s revision request. Exact English item wording for the analyzed questions is reproduced below. The study used a bilingual Arabic-English questionnaire. To avoid transcription error, the exact verbatim Arabic fielded wording is not reconstructed here; the translation, back-translation, and cognitive-testing process is described in the Methods section.

Source note: Study-developed questionnaire adapted from the screen-time literature for cultural relevance in the UAE. Credits: Ola Mansour Tarkhoun, Maryam Omar Owaid, Tasnime Brinsi, Rooa Fayez Bin Kuwaiyer, Aya Bshar Kanani, Salsabila Brinsi, Yusuf Pervez.

**Table 5:** Eligibility and demographic items

Predictor	IRR	95% CI	Wald z	p-value
Screen time (hours/day)	1.031	1.001-1.062	2.04	0.041
Bedroom screen use (yes)	1.3	1.130-1.495	3.67	<0.001
Age group (ref = 11-15 years)				
4-6 years	1.077	0.901-1.286	0.82	0.414
7-10 years	1.029	0.875-1.210	0.34	0.731
1-3 years	0.725	0.544-0.955	-2.24	0.025
Gender (ref = male)				
Female	1.004	0.883-1.141	0.06	0.95
Parent nationality (ref = expatriate)				
UAE national	1.111	0.953-1.293	1.36	0.175
Household income (ref = >20,000 AED)				
<5,000 AED	0.9	0.698-1.149	-0.83	0.405
10,000-20,000 AED	0.914	0.779-1.071	-1.11	0.267
5,000-10,000 AED	0.941	0.789-1.119	-0.69	0.492

**Table 6:** Screen time and usage

Item	English/original text	Arabic/translated text	Response options/notes
Q6	On average, how many hours per day does your child spend on screens (TV, smartphone, tablet, computer, video games)?	في المتوسط، كم عدد الساعات التي يقضيها طفلك يوميًا أمام الشاشات (التلفاز، الهاتف الذكي، الجهاز اللوحي، الكمبيوتر، ألعاب الفيديو)؟	Less than 1 hour; 1-2 hours; 3-6 hours; 6-8 hours; more than 8 hours.
Q7	What types of screen activities does your child engage in most frequently?	ما أنواع الأنشطة التي يقوم بها طفلك على الشاشة بشكل أكثر تكرارًا؟	Select all that apply: watching cartoons/videos; playing video games; using educational apps; social media (YouTube, TikTok, Instagram, etc.).
Q8	When does the child use screens the most?	متى يستخدم الطفل الشاشات أكثر؟	Morning; afternoon; evening; before bedtime.
Q9	Where does your child usually use screens?	أين يستخدم طفلك الشاشات عادةً؟	Select all that apply: bedroom; living room; school; car/traveling; while eating.
Q10	Does the child use screens more on weekdays or weekends?	هل يستخدم الطفل الشاشات أكثر في أيام الأسبوع أم عطلات نهاية الأسبوع؟	More on weekdays; more on weekends; about the same.
Q11	At what age did the child start using screens regularly?	في أي عمر بدأ الطفل باستخدام الشاشات بشكل منتظم؟	Below 1 year; 1-3 years; 4-6 years; 7 years or older.
Q12	Do you set time limits on your child’s screen use?	هل تضع حدودًا زمنية على استخدام طفلك للشاشة؟	Yes; no.
Q13	Do they have their own devices?	هل لديهم أجهزتهم الخاصة؟	Yes; no.

**Table 7:** Behavioral changes

Item	English/original text	Arabic/translated text	Response options/notes
Q14	Have you noticed any behavioral changes in your child related to screen time? Please specify (select all that apply).	هل لاحظت أي تغييرات سلوكية في طفلك مرتبطة بوقت الشاشة؟ يرجى التحديد (اختر كل ما ينطبق).	Select all that apply: increased irritability or tantrums; decreased attention span; increased hyperactivity; decreased physical activity; difficulty sleeping or nightmares; social withdrawal or reduced interaction with family; increased aggression; anxiety or mood swings; no significant changes noticed. For analysis, the first eight options were coded as dichotomous symptom indicators (selected = yes; not selected = no).
Q15	Has the child ever expressed frustration, anger, or restlessness when screen time is limited or removed?	هل عبر الطفل يومًا عن الإحباط أو الغضب أو القلق عندما يتم تقييد وقت الشاشة أو إزالته؟	Yes, frequently; yes, sometimes; no, rarely; no, never.
Q16	How would you describe your child’s attention span?	كيف تصف مدى انتباه طفلك؟	Very short; somewhat short; average; above average.
Q17	Has your child’s interest in outdoor play and social activities decreased due to screen use?	هل انخفض اهتمام طفلك باللعب في الهواء الطلق والأنشطة الاجتماعية بسبب استخدام الشاشات؟	Yes; no.
Q18	How has screen time affected the child’s social interactions?	كيف أثر وقت الشاشة على تفاعلات الطفل الاجتماعية؟	More engaged with family and friends; less interested in social interactions; no noticeable impact.
Q19	Has online learning influenced your child’s behavior or social interactions?	هل أثر التعلم عبر الإنترنت على سلوك طفلك أو تفاعلاته الاجتماعية؟	Yes, positively; yes, negatively; no significant impact; not sure.
Q20	Has the child’s academic performance been affected by screen time?	هل تأثرت التحصيل الدراسي للطفل بوقت الشاشة؟	Yes, it has improved; yes, it has declined; no significant impact.

**Table 8:** Parental perspectives and control measures

Item	English/original text	Arabic/translated text	Response options/notes
Q21	Do you believe screen time negatively affects your child’s behavior?	هل تعتقد أن وقت الشاشة يؤثر سلبًا على سلوك طفلك؟	Yes; no; not sure.
Q22	What strategies do you use to manage your child’s screen time? (Select all that apply)	ما هي الاستراتيجيات التي تستخدمها لإدارة وقت استخدام طفلك للشاشة؟ (اختر كل ما ينطبق)	Select all that apply: setting time limits; encouraging alternative activities; using parental control apps; co-viewing content with the child.
Q23	What do you think is the biggest challenge in reducing screen time for your child?	ما رأيك في أكبر تحدٍ في تقليل وقت الشاشة لطفلك؟	Lack of alternative activities; peer pressure (friends/schoolmates use screens); my child refuses to engage in non-screen activities; work commitments prevent me from monitoring screen time.
Q24	What do you prefer?	ماذا تفضل؟	Online-facilitated teaching methods; traditional teaching methods; mix of both.
Q25	Do you believe children’s screen time should be monitored more strictly in schools?	هل تعتقد أنه ينبغي مراقبة وقت شاشة الأطفال في المدارس بشكل أكثر صرامة؟	Yes; no; not sure.
Q26	What do you think is an appropriate daily screen time limit for children?	ما رأيك في الحد اليومي المناسب لاستخدام الشاشات للأطفال؟	Less than 1 hour; 1-2 hours; 3-4 hours; no limit, as long as content is appropriate.
